# Mutated p53 in HGSC—From a Common Mutation to a Target for Therapy

**DOI:** 10.3390/cancers13143465

**Published:** 2021-07-10

**Authors:** Aya Saleh, Ruth Perets

**Affiliations:** 1 Ruth and Bruce Rappaport Faculty of Medicine, Technion—Israel Institute of Technology, Haifa 3109601, Israel; sayahs@campus.technion.ac.il; 2 Division of Oncology, Clinical Research Institute at Rambam, Rambam Medical Center, Haifa 3109601, Israel

**Keywords:** *TP53*, mutated p53, epithelial ovarian cancer, high grade serous ovarian cancer, targeting mutated p53, gain of function

## Abstract

**Simple Summary:**

Ovarian high-grade serous cancer (HGSC), the most common and the deadliest subtype of epithelial ovarian cancer, is characterized by frequent mutations in the *TP53* tumor suppressor gene, encoding for the p53 protein in nearly 100% of cases. This makes p53 the focus of many studies trying to understand its role in HGSC. The aim of our review paper is to provide updates on the latest findings related to the role of mutant p53 in HGSC. This includes the clinical outcomes of *TP53* mutations in HGSC, upstream regulators and downstream effectors of p53, its function in the earliest stages of HGSC development and in the interplay between the tumor cells and their microenvironment. We summarize with the likelihood of p53 mutants to serve as biomarkers for early diagnosis and as targets for therapy in HGSC.

**Abstract:**

Mutations in tumor suppressor gene *TP53,* encoding for the p53 protein, are the most ubiquitous genetic variation in human ovarian HGSC, the most prevalent and lethal histologic subtype of epithelial ovarian cancer (EOC). The majority of *TP53* mutations are missense mutations, leading to loss of tumor suppressive function of p53 and gain of new oncogenic functions. This review presents the clinical relevance of *TP53* mutations in HGSC, elaborating on several recently identified upstream regulators of mutant p53 that control its expression and downstream target genes that mediate its roles in the disease. *TP53* mutations are the earliest genetic alterations during HGSC pathogenesis, and we summarize current information related to p53 function in the pathogenesis of HGSC. The role of p53 is cell autonomous, and in the interaction between cancer cells and its microenvironment. We discuss the reduction in p53 expression levels in tumor associated fibroblasts that promotes cancer progression, and the role of mutated p53 in the interaction between the tumor and its microenvironment. Lastly, we discuss the potential of *TP53* mutations to serve as diagnostic biomarkers and detail some more advanced efforts to use mutated p53 as a therapeutic target in HGSC.

## 1. Epithelial Ovarian Cancer

Epithelial ovarian cancer is the deadliest malignancy of the female reproductive system and the fifth leading cause of cancer deaths among women in the United States, accounting for nearly 5% of all tumors in women [1]. For 2021, 21,410 new incidents and 13,770 deaths were estimated in the United States [1]. Globally, ovarian cancer (OC) is the eighth most common cancer and eighth most common cause of cancer-related mortality in the female population [2].

Several risk factors have been linked to ovarian cancer development. Hysterectomy, pregnancy, parity, breastfeeding, tubal ligation, female sterilization, and oral contraceptive use likely reduce the risk of ovarian cancer. On the other hand, inheritable mutations in BRCA1/2 genes, infertility, early menarche, late age at menopause (usually over the age of 50), hormonal therapy, exposure to specific environmental factors (e.g., talc, pesticides, and herbicides), as well as lifestyle factors such as smoking, obesity and unhealthy diet, are all associated with an increased risk to develop ovarian cancer [3,4].

EOC is not considered a single disease entity, but rather represents a group of complex and heterogeneous diseases [5]. EOC tumors are classified into four major histologic types: serous (~70%), endometrioid (10%), clear cell (10%), and mucinous (3–10%) [6]. These differ in cellular origin, molecular changes, and in their potential for targeted therapy [7]. However, three of these morphologically different types of EOC (serous, endometrioid, and clear cell) are currently treated the same [7,8], for lack of approved subtype specific therapy. 

Based on histopathological and molecular features, a dualistic model of carcinogenesis was suggested for EOC that broadly divides epithelial ovarian tumors into two groups: type I and type II. Type I tumors are less frequent, accounting for only 5–10% of all epithelial ovarian tumors, growing slowly, less likely to spread, and usually diagnosed at early stages. Type I includes low-grade serous, endometrioid, clear cell, and mucinous ovarian carcinomas as well as Brenner tumors, which likely evolve gradually from borderline tumors [9,10,11,12]. In addition, these tumors typically have specific mutations in KRAS, BRAF, ERBB2, CTNNB1, PTEN, PIK3CA, ARID1A, and PPPR1A genes, and are considered relatively chromosomally stable with rare mutations in *TP53*, the gene coding for the tumor suppressor p53. Type II tumors are much more frequent and aggressive than type I, tend to grow more rapidly, present at an advanced stage, have a very high frequency of *TP53* mutations, but rarely harbor the mutations characterizing type I tumors. Type II tumors also have molecular alterations that disrupt expression of BRCA1/2 either by mutation of the gene or promotor methylation. Furthermore, about 50% of type II tumors harbor defects in homologous recombination DNA repair pathways. Another feature of these tumors is they are genetically highly unstable, with widespread DNA copy number variations, including amplifications or deletions of various genes [10,12,13]. Type II tumors include high-grade serous carcinoma, which is the vast majority of tumors, high-grade endometrioid carcinoma, malignant mixed Müllerian tumors (carcinosarcoma), undifferentiated carcinomas, and some clear cell carcinomas, with no recognizable precursors detected in the ovary [12].

EOC carries a poor prognosis with an expected 5- and 10-year survival rate of approximately 30–35% and 15%, respectively [7,14,15,16]. The main reason for the low survival rate is that early ovarian cancer usually has no or very subtle clinical symptoms, leading to 60% of ovarian cancers being diagnosed at advanced stages, carrying very low chances of cure. 

The standard treatment for EOC patients involves surgery and a combination of platinum and paclitaxel-based chemotherapies. The initial response to chemotherapy is largely satisfying, which in most cases leads to complete tumor remission; however, in nearly 70% of women, the disease recurs during the first three years [4] with no efficient treatment options. Despite significant research over the past decades, no remarkable advancement has occurred regarding the development of novel and effective therapeutic agents against EOC. Currently, targeted therapies are still limited and the overall survival rate of patients with HGSC remains low. Polyadenosine diphosphate-ribose polymerase (PARP) inhibitors and the vascular endothelial growth factor (VEGF) inhibitor Bevacizumab are currently the only approved targeted therapies against HGSC. Despite these new therapies, the grim prognosis of this disease is largely unchanged [17,18], emphasizing the urgent unmet need for developing more efficient therapeutics for treating this fatal type of EOC and enhancing patients’ survival rate.

In this article we review the major role of *TP53* in HGSC and present the potential of targeting p53 as a therapeutic alternative for this disease

## 2. p53 Function in Normal Non-Cancerous Cells

**Figure 1:**
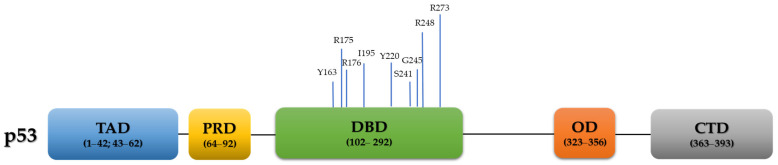
p53 domains and the frequency of the nine most common hotspot mutations of the human TP53 gene in HGSC, based on the TCGA data [13,23]. TAD: transactivation domain; PRD: proline-rich domain; DBD: DNA binding domain; OD: oligomerization domain; CTD: C-terminal domain.

*TP53*, a well-established and extensively studied tumor suppressor gene, is located in chromosome 17p13.1 of the human genome [19] and composed of 11 exons, encoding the 53KDa protein p53. Structurally, it is a homo-tetrameric protein, consisting of 393 amino acids and containing four functional domains: 1. Two N-terminal transcriptional activation domains (TAD, residues 1–42 and residues 43–62), 2. A proline-rich domain (PRD, residues 64–92), 3. Central sequence-specific DNA binding domain (DBD, residues 102–292), 4. Oligomerization domain (OD, also known as tetramerization domain; resides 323–356), and 4. C-terminal regulatory domain (residues 363–393) [20,21,22] (Figure 1).

p53 plays a fundamental role in controlling cell homeostasis and keeping genome integrity via regulating diverse biological processes in response to stress, including cell cycle arrest, apoptosis, ferroptosis (a special type of cell death caused by reactive oxygen species), DNA damage repair, senescence, and metabolism [24,25,26,27]. p53 executes most of its roles by acting as a sequence-specific DNA binding transcription factor that regulates expression of a wide range of coding and non-coding genes [28,29,30]. However, like other transcription factors, several studies unveiled that p53 actions and localization are not confined to the nucleus. One well characterized example is the ability of p53 to exert its activities, such as apoptosis induction, while localized in the cytoplasm. Moreover, p53 also functions in a transcription-independent way [30].

Under normal un-stressed conditions, wild type p53 has a short half-life time and is kept at low protein levels in the cell, constantly subjected to proteasomal degradation by its E3 ubiquitin ligase MDM2 (murine double minute2) [31,32,33,34]. Under stressed conditions, either external to the cell or internal, such as hypoxia, abnormal proto-oncogene activation, DNA damage (e.g., exposure to UV or gamma irradiations), mitogenic signaling, cellular ribonucleotide depletion, mitotic spindle damage or nitric oxide (NO) production [35,36], p53 is stabilized and becomes active. p53 function is regulated via activation, degradation, and intracellular translocation, which are all regulated by many post-translational modifications (PTMs), such as phosphorylation, acetylation, ubiquitination, and methylation [37,38,39,40]. For example, it has been reported that during DNA damage, phosphorylation of p53 at numerous sites leads to disruption of the MDM2-p53 association complex and consequently to p53 protein stabilization and activation [31]. Stable p53 is translocated to the nucleus where it performs its well-known aforementioned biological functions [27,41,42] by binding DNA, where it can interact with other transcriptional regulatory proteins [43] to enhance transcription of various critical genes, including CDKN1A, *PCNA*, *GADD45,* *BAX,* and MDM4 [44], to either promote anti-tumorigenic or suppress pro-tumorigenic effects.

Moreover, p53 activity is indirectly controlled by several mechanisms that regulate MDM2, and therefore disrupt p53-MDM2 interaction. These include PTMs, physical sequestration, and degradation of MDM2 [45,46].

Additional E3 ubiquitin ligases are involved in p53 ubiquitination and degradation or changing p53 sub cellular localization such as ARF-BP1/Mule, COP1, Pirh2 and MSL2 [47,48,49,50]. Additionally, MDMX (also known as MDM4), a homolog of MDM2, and other viral proteins (SV40 large T-antigen, adenovirus E1B-55-kDa protein, and the E6 oncoprotein of human papilloma virus (HPV) types 16 and 18) have been implicated in inhibiting the wild-type activity of p53 [51,52].

## 3. *TP53* Mutations in Cancer

*TP53* is the most frequently mutated tumor-suppressor gene in cancers, found in over 50 percent of all human tumors [19,53,54,55]. However, different tumor types have different *TP53* mutations profiles [54]. In general, *TP53* mutations are categorized into several groups based on their location, type of mutation, and phenotype of the resulting mutant p53 protein. The DNA binding domain (ranging from exon 4–8) is the most common site of *TP53* mutations [13], containing approximately 80 percent of *TP53* mutations [56]. However, many mutations have been detected outside this region [57], spanning the majority of the *TP53* gene codons [8]. Mutation types in *TP53* include missense, nonsense, frameshift, in-frame insertions or deletions (INDEL), and splice site, all of which are common in human cancers, particularly in ovarian cancers [13,55,58,59]. The type or site of a certain mutation can change the functional consequences of p53 mutants [23]. For instance, it has been suggested that a different phenotype is obtained from frameshift mutations as compared to missense ones. While some of *TP53* missense mutations yield full-length p53 protein with extended half-life accumulation of inactive protein, frameshift mutations do not typically lead to p53 accumulation, and nonsense mutations usually produce a truncated unstable protein [60].

Mutations in *TP53* can give rise to different phenotypes; mainly gain-of-function (GOF) and loss-of-function (LOF). GOF mutp53 can be obtained from *TP53* variations that not only abolish the wild-type properties of p53, but also endow the mutant protein with new oncogenic activities [22], contributing to malignant progression and resistance to anti-cancer therapies [59,61,62]. These mutants usually result from missense mutations that lead to overexpression, high stabilization, and nuclear p53 protein accumulation [26,58]. Importantly, not all accumulating p53 mutants are evidenced as GOF [56], and gain of function phenotype is tissue dependent [63]. LOF p53 mutants are usually obtained from either nonsense or frameshift mutations that significantly perturbate the translated protein. The main outcome of LOF mutants is the complete loss of wild-type p53 tumor suppressive activity. Dominant negative (DN) effects can be exerted by a mutant p53, where the mutated allele binds to the WT one and masks or prohibits its activity, especially if the mutant protein is excessively expressed over the WT one [59,62,64]. Silent mutations (those that do not change the amino acid sequence) are categorized as WT mutations. Despite being considered neutral, the high prevalence of silent mutations, and some evidence of correlation of silent p53 mutants with poor survival, raise the question whether silent p53 mutations are in fact neutral [65]. The remaining single base substitutions in *TP53* with unknown or undefined function, neither GOF nor LOF, are designated as "unclassified" mutations [66]. 

The majority (~70%) of all cancer-associated *TP53* alterations are missense mutations caused by a single amino acid substitution in the protein coding sequence [13,67,68,69], mostly in amino acids residing in the highly conserved DBD. Six of them are referred to as ‘hotspot’ residues: R175, G245, R248, R249, R273, and R282 [70,71] that usually have high frequencies of occurrence (5.1%, 3.3%, 7%, 2.9%, 6.7% and 2.9%, respectively) [26,72]. These ‘hotspot’ codons usually possess a GOF property, in comparison to their WT counterparts [26,73,74]. These missense ‘hotspot’ residues are further sub-classified into contact (functional) and conformational (structural) groups. Contact mutations (i.e., R248 and R273) influence direct p53 binding to DNA, consequently altering the capacity to *trans*-activate promoters of its target genes, whereas structural mutations, such as R175, G245, R249 and R282, produce structurally unstable and partially unfolded p53, which perturbates its activity [26,75,76]. Nevertheless, the segregation between the two groups is not absolute [77]. Other known structural *TP53* mutations include ‘non-hotspot’ codons, such as P250L, E258V, R110L, and R100P. Of note, while DNA contact mutants are accompanied with exclusive p53 nuclear staining, structural mutants display cytoplasmatic p53 localization [78].

## 4. *TP53* Mutations in HGSC and Their Clinical Relevance

Somatic mutations in *TP53* are the major molecular characteristic of HGSC, where *TP53* is altered in at least 96% of HGSC tumors [13,56,79,80]. Particularly, in about 60–70% of HGSC cases, these mutations preserve the protein resulting in its overexpression and nuclear accumulation, mostly because of missense mutations, specifically in the DBD, whereas protein expression is lost in up to 39% of p53 [13,81] due to nonsense mutations that create premature stop codon, resulting in an unstable protein [60]. 

Based on the TCGA dataset, the most frequent types of *TP53* variations in HGSC are missense (60.52%), followed by frameshift (15.24%), nonsense (10.73%), splice site (10.52%), and in-frame mutations (3.22%) [13]. A total of 126/282 (44.68%) hotspot mutations were observed in HGSC according to the TCGA, with the nine most common ones being: R273 (20.63%, 26/126), R248 (16.67%, 21/126), R175 (14.29%, 18/126), Y220 (9.52%, 12/126), I195 (9.52%, 12/126), C176 (8.73%, 11/126), G245 (8.73, 11/126), S241 (6.35%, 8/126), and Y163 (6.35%, 8/126) [23] (Figure 1). Interestingly, while many driver mutations evolved in response to treatment leading to treatment resistance [82], *TP53* mutations remain unchanged during the course of the disease [83]. 

The high prevalence of p53 mutations in HGSC leads to p53 mutations being a surrogate for a diagnosis of HGSC, which differentiates it from other EOC subtypes. Next generation sequencing to detect p53 mutations is not a straightforward method to use in clinical practice, and therefore p53 immunohistochemistry is a convenient alternative technique. Two studies have shown that very high or no p53 stain suggests, with high sensitivity, that the tumor sample carries mutant p53 [56,84]. 

In addition to the diagnostic utility of mutated p53 detection, the prognostic and predictive roles of specific *TP53* mutations are being studied, with recent discordant findings on the association of GOF-mutated *TP53* type with platinum treatment resistance [23,66,69,85]. Based on an analysis of the TCGA dataset of ovarian HGSC cases, one study reported that patients with GOF *TP53* are more likely to be platinum-resistant and develop distant metastasis as compared to HGSC patients with no evidence of GOF, with no difference in progression-free survival (PFS) or overall survival (OS) between patients with GOF or non-GOF *TP53* mutations [69]. Analysis of the same data by another research group showed that in patients with advanced serous ovarian carcinomas, GOF *TP53* mutations predicted worse prognostic outcomes; particularly, resistance to chemotherapy and high risk of recurrence. In that study, patients with GOF *TP53* mutations had a significantly shorter PFS [66]. A more recent study [85] reported that tumors carrying GOF *TP53* mutations were more susceptible to platinum therapy, contradicting the two previous studies based on the TCGA [66,69]. Perhaps a recent study by Tuna et al. resolves these contradictory studies, showing that each mutation behaves differently and has distinct effects on survival regardless of the type, structural classification, or location of the mutation [23]. This suggests that the previous analyses of GOF mutations as a class might be simplistic, and heavily dependent on the composition of mutations in each study. 

Most of the studies conducted on aberrant p53 role in cancers have concentrated on its full-length isoform. However, at least 13 different isoforms are encoded by *TP53* through different mechanisms: alternative promoter usage, alternative splicing, and alternative translation start sites [86,87,88]. Δ133p53 is an amino-terminal truncated *TP53* isoform resulting from alternative promoter usage that was shown to be highly expressed in HGSC samples, and correlate with longer OS [89]. 

For many years, since the publication of the TCGA [13], a debate existed whether WT p53 HGSC exists, or the WT p53 cases in the TCGA were a misclassification [90]. However, a recent comprehensive report from the MSK-IMPACT platform showed that WT p53 HGSC tumors do exist and comprise 2.5% of the 987 HGSC samples tested. Of these, 40% of tumors showed low-grade serous carcinoma-like features, and 60% showed clear HGSC characteristics and down regulation of WT p53 either by reduced protein expression and/or MDM2 amplification [91]. 

## 5. Regulation and Function of p53 in HGSC

### 5.1. Upstream Regulators of p53 in HGSC

**Figure 2:**
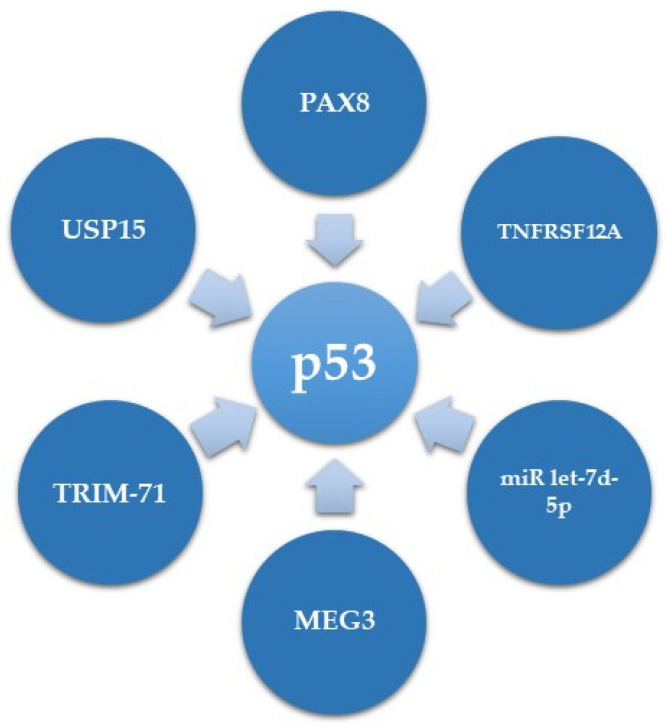
Upstream regulators of p53.

While several downstream mediators of mutant p53 are known [22,26], much less is known regarding its upstream regulators in HGSC. We will detail herein the current information on p53 expression regulators in HGSC and summarize this information in Table 1 and Figure 2.

In terms of transcriptional regulation of mutant p53 expression, our research group has recently demonstrated that in ovarian HGSC, PAX8, which plays an oncogenic role, can directly bind to the promoter of GOF mutp53, and positively regulate its expression. Consequently, this results in overexpression of CDKN1A (coding for p21), a *TP53* target gene in HGSC, which has a non-canonical cytoplasmic role, leading to increased proliferation and decreased apoptosis of HGSC cells [92]. 

However, most of the p53 expression level regulation in HGSC is regulation of protein stability. One example is a study showing that Fibroblast growth factor-inducible 14 (Fn14, encoded by TNFRSF12A) can induce chemotherapy sensitivity in OVCAR3 HGSC cells, bearing the *TP*53 R248Q mutation. This function of Fn14 is mediated via enhancement of MDM2 mediated ubiquitination and degradation of p53 [93].

Several other proteins have been shown to affect the degradation of mutant p53. One of them is the E3 ubiquitin ligase TRIM-71 (Tripartite motif containing 71, also known as LIN41) that induces degradation of p53 mutants in HGSC [94]. Accordingly, the expression of TRIM-71 is downregulated in several cancers including ovarian carcinoma [98]. An opposite role is mediated by the deubiquitinase USP15 (Ubiquitin Specific Protease 15), which is overexpressed in several cancers, including ovarian carcinoma [99]. USP15, by deubiquitination, selectively elevates levels of the highly stable and aggregated GOF p53 R175H mutation, while having minimal to no effect on WT p53 and R273H mutants [95]. This suggests that different p53 mutants can have different regulation methods in HGSC. 

Accumulating evidence have demonstrated that non-protein coding sequences, such as lncRNAs, miRs and circRNAs, are implicated in various biological and cellular events, including tumorigenesis [100,101,102]. MEG3 (maternally expressed gene 3), encoding an approximately 1.6 Kb lncRNA, was shown to upregulate mutant p53 RNA expression as well as protein expression and activity, albeit in an unknown mechanism [96]. Regarding miRs, it was recently demonstrated that in ovarian cancer miR let-7d-5p targets the expression of high mobility group A (HMGA) protein, which in turn affects mutant p53 expression in the mRNA and at the protein level [97]. However, the mechanisms by which miR let-7d-5p and HMGA affect mutant p53 expression levels are unclear. 

### 5.2. Downstream Effectors of Mutant p53 in HGSC

**Figure 3:**
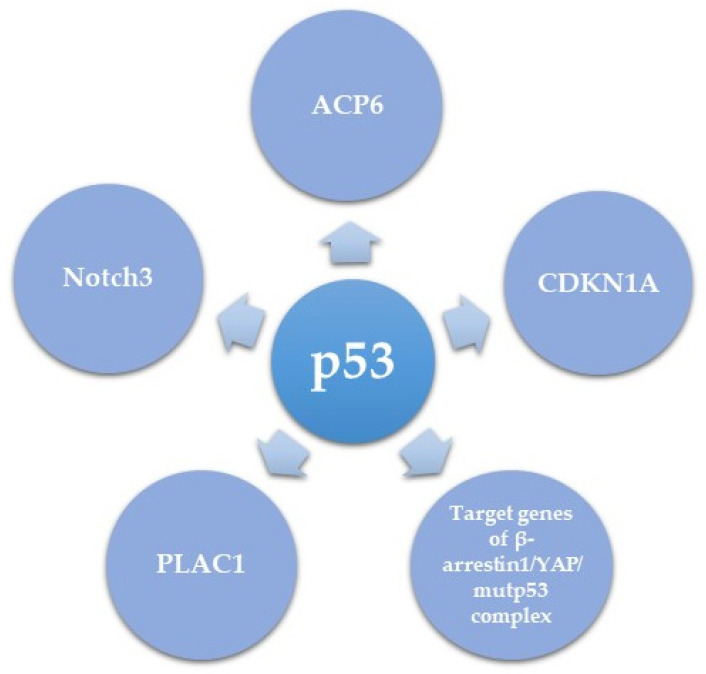
Downstream mediators of mutant p53 function.

p53 mutations often impair the protein’s ability to activate downstream target genes of the WT protein, but also gain new oncogenic functions. These new oncogenic activities can be mediated either by the dominant negative effect of mutant p53 on the activity of WT p53, or via a new GOF activity of p53 [103]. Herein we summarize several downstream mediators of the role of mutant p53 in HGSC (Table 2 and Figure 3) and try to understand whether novel mutant p53 transcriptional targets are shown, or mutant p53 acts as an antagonist to the role of WT p53. The first downstream target of mutant p53 is PLAC1 (encoding placenta-specific-1), an oncogene involved in proliferation, invasion, and metastasis of cancer cells [104,105]. PLAC1 has been shown to be transcriptionally inhibited by WT p53 [106] and in HGSC this inhibition is de-repressed by mutant or null p53. The oncogenic role of PLAC1 suggests that induction of PLAC1 expression could be a mechanism of the oncogenic role of mutant p53 in HGSC [107]. Another pro-proliferative and pro metastatic target of mutant p53 is Notch3, which in turn activates CCNG1, the latter having a pro-metastatic effect on EOC in vivo [108]. It is unclear whether the effect of mutant p53 on Notch signaling occurs via inhibition of the activity of WT p53, or whether this is a true GOF of WT p53. 

Recently, Tocci and colleagues elegantly unveiled a critical role of β-arrestin1/YAP/mutant p53/TEAD complex in HGSC. The study demonstrated that upon endothelin-1 receptor (ET-1R) signaling induction, β-arrestin1 interacts with YAP, stimulating its nuclear shuttling. Once in the nucleus, β-arrestin1 allows the binding of YAP to mutp53, after which they are recruited with TEAD to YAP/mutp53 target gene promoters in order to regulate their transcription. This consequently results in enhanced proliferation, survival, and invasion of HGSC cell lines as well as metastasis in patient-derived xenograft models. Interestingly this manuscript clearly shows a GOF role to mutant p53 as part of a transcriptional complex, without involvement of WT p53, suggesting a new GOF role for mutant p53 [109].

Dysregulated metabolism is another hallmark of HGSC progression [111]. Chryplewicz et al. recently showed that the R237H mutant p53 dependent transformation of FTSEC led to downregulated expression of lysophosphatidic acid (LPA) degradation enzyme, lysophosphatidic acid phosphatase type 6 (ACP6), resulting in high LPA levels. High LPA levels led to phosphorylation of the tight junction proteins Paxillin and FAK, contributing to the invasive properties of the cells. Furthermore, down regulation of ACP6 in vitro and in vivo in a xenograft mouse model led to an aggressive pro-migratory phenotype, while overexpression of ACP6 led to reduced proliferation. In order to correlate with human tumors, the authors of this manuscript analyzed three human HGSC tumors and adjacent normal fallopian tube cells, and found an inverse correlation between mutant p53 expression and APC6 correlation, further supporting the negative regulation of ACP6 by mutant p53. This suggests that down-regulation of ACP6 is another mechanism of the oncogenic function of mutated p53. Of note, the three hotspot p53 mutations R175H, R249S, and R273H all led to the same effect of ACP6 down regulation, albeit R273 had the most pronounced effect. It is unclear whether the effect of the three mutants on ACP6 expression is direct or via transcriptional regulation of a common network, that in turn downregulates ACP6 expression [110]. 

Taken together, mutant p53 activates a complex downstream network that affects many hallmarks of HGSC progression including cancer metabolism, invasion, and metastasis, altogether promoting cancer progression. 

## 6. The Role of p53 in HGSC Pathogenesis

*TP53* mutations are the earliest and most frequent genetic alteration in HGSC. Herein we will summarize current data regarding the role of p53 in the pathogenesis of HGSC. 

Accumulating evidence emerging from ovarian cancer research over the past two decades strongly indicate that most HGSCs are derived from the secretory epithelial cells of the fallopian tube fimbria [112,113,114,115], rather than from the ovarian surface epithelium (OSE) or the cortical inclusion cysts, which were originally considered the site of origin [116,117,118,119]. This paradigm shift has gained wide acceptance and is based on the detection of early lesions, designated serous tubal intraepithelial carcinomas (STIC), found in the fallopian tube of women at high-risk for developing serous carcinomas, as well as in patients with disseminated HGSCs [120,121,122,123,124,125,126,127,128]. The capability of normal fallopian tube epithelium (FTE) to transform into HGSC has also been proven by in vitro and in vivo studies [129,130,131]. 

A detailed and comprehensive description of HGSC development from the FTE has been reported. The normal fallopian tube is composed of two types of epithelial cells: secretory and ciliated. The earliest clearly recognizable step of transformation is the ‘p53 signature’. The p53 signature is entirely comprised of short stretches of continuous normally looking monolayered secretory epithelial cells showing nuclear p53 overexpression and positive ϒH2AX staining representative of DNA damage. The p53 signature cells most likely originates in stem/progenitor cells after clonal expansion [132], carrying a stabilizing missense p53 mutation. p53 signatures have no proliferative capacity as indicated by the lack of Ki-67 staining, a known marker for proliferation [122]. The next step of transformation, STIC, retains *TP53* mutation as well as strong nuclear p53 expression and DNA damage, but also acquires new properties: increased proliferation, nuclear pleomorphism, and loss of epithelial polarity. Ultimately, when STIC gains invasive features, it becomes HGSC [133], and early on metastasizes to the ovary and then to other pelvic organs [115,134]. Several studies have revealed that FT lesions, including the ‘p53 signature’, STIC lesion, and invasive HGSC from the same patient shared an identical *TP53* mutation, supporting a clonal relationship between the precursor lesions and the invasive cancer [121,122,135,136,137,138,139]. 

The finding of p53 mutations in nearly all HGSC samples [13], together with its early mutation in HGSC pathogenesis, suggests that p53 acts as a driver gene in HGSC, and its dysfunction is a necessity for developing the genomic instability characterizing this disease [140]. Nevertheless, mutation of *TP53* alone is insufficient for FTE transformation and HGSC formation [141].

As mentioned earlier, the most widespread *TP53* mutations in HGSC are missense (~60%), whereas nonsense mutations appear in the remaining cases [122,136]. Missense mutations are associated with strong diffusion of p53 staining in STICs and HGSCs (‘p53 signature’); however, complete lack of p53 immunoreactivity is correlated with nonsense mutations yielding a truncated protein which is undetectable by p53 antibody and is referred to as ‘p53 null mutation’ [136]. Noteworthy, a significant portion (20–50%) of STIC lesions can be negative for p53 immunostaining [127]. To improve the diagnostic accuracy of STIC, especially for p53 null STICs, Novak et al. suggested the use of STMN1 (encoding Stathmin 1) and p16 as sensitive and specific adjunct biomarkers along with p53 and Ki-67 [142]. More recently, a case report proposed that STICs expressing γ-H2AX, the well-known marker of DNA damage, without p53 overexpression may be a potential candidate of null-type *TP53*-mutated FT cells, and are termed “γ-H2AX responsive foci” [143].

The cause of *TP53* mutation is unknown but it was proposed to be highly linked to ovulation [132], as the number of lifetime ovulation cycles has been shown to positively correlate with the probability of developing ovarian tumors overexpressing mutant p53 [144,145]. Furthermore, epidemiological research demonstrated that ovulation inhibition by oral contraceptive usage reduced risk of EOC development in short- and long-term settings [146,147].

Not much is known regarding the role of *TP53* mutations in HGSC precursor lesions. Utilizing a model of murine oviduct epithelia (the equivalent of human fallopian tube) bearing a *TP53* mutation, Quartuccio et al. showed that mutant p53 drives migration of FTE cells via upregulating *Snai2* transcription, the gene encoding Slug protein. Slug increases the expression of its downstream target Vimentin, a mesenchymal marker, leading to EMT promotion. This study was the first to explore a function of *TP53* mutation in FTE. Intriguingly, no increase in cell migration of OSE carrying a similar *TP53* mutation was noted, designating that this mutation effect is cell-type specific. These findings contribute to the understanding of the high metastatic potential of high-grade serous tumors [141]. Another clue to the selective role of mutant p53 in FTE vs. OSE comes from a study showing that in vitro, using mouse immortalized cells, CHD6 (encoding cadherin-6 type 2) expression is directly repressed by GOF mutp53 in the mouse oviductal cells, but not in mouse ovarian surface epithelium cells. Similarly, CDH6 is expressed in human FTE but was not detected in human OSE [148].

Very recently, it has been demonstrated that acquisition of specific common GOF *TP53* mutations (R273H, R248 and R175, in FTE cells) induce the tropomyosin receptor kinase B (TrkB) transcription or recycling, leading to its increased oncogenic activity. This subsequently enhances responses of FTE to ovarian BDNF (brain-derived the neurotrophic factor) secreted to the follicular fluid and present in distal tubal microenvironment, contributing to survival, migration, and attachment of FTE tumor precursors. The study implicates the synergism of environmental cues, such as growth factors of the ovary with *TP53* mutations in the FT, to encourage transformation of FTE towards invasive HGSC [149].

## 7. The Role of p53 in HGSC Microenvironment 

A supportive tumor microenvironment enables some of the emerging hallmarks of cancer progression [150]. The cancer stroma is a major part of the tumor microenvironment that is believed to play a significant role in tumor behavior, such as invasion, metastasis, and response to therapy [151]. Several studies demonstrated that loss of p53 function in stromal cells was correlated with poor prognosis and high tumor relapse [152,153,154]. More recent research has shown that when normal fibroblasts turn into cancer associated fibroblast (CAFs) the transcriptional program of WT p53 changes from a tumor suppressive program to a tumor supportive one. Furthermore, Schauer et al. showed that elevated expression and secretion of interleukin-1β (IL-1β) from EOC cells, and overexpression of its receptor IL-1R1 in cancer-associated fibroblasts (CAFs), resulted in attenuated p53 expression in CAFs. Knockdown of p53 in ovarian fibroblasts resulted in activation of a pro-tumorigenic inflammatory response in vivo [155]. Similarly, stromal fibroblasts lacking p53 function induced invasiveness in an organotypic model of p53 knockdown CAFs and immortalized ovarian epithelial cells (T72), but not in a similar co-culture with control CAFs. This effect was proposed to be mediated by reactive nitrogen species (RNS)-induced cytokine ICAM1 secretion. Of note, the effect of p53 deficient CAFs on immortalized fallopian tube secretory epithelial cells was not tested [156]. 

Mutated p53 and the type of specific mutations, missense vs. nonsense, dictate the cancers cell’s response to signals from the microenvironment. One such example was shown a decade ago, when Hwang et al. described a stromal-epithelial interaction in ovarian carcinogenesis related to alterations in p53/miR-34/MET network [157]. MET, a receptor tyrosine kinase activated by hepatocyte growth factor (HGF), is known to play a critical role in metastasis [158,159]. In normal ovarian epithelial cells, WT p53 represses MET transcription directly via binding its promoter and/or indirectly through recruiting miR-34, thus abolishing MET-induced cell motility and invasion. However, when *TP53* is mutated, it is still capable of binding the MET promoter, but the regulation through miR-43 is absent, leading to mutated p53 retaining the repression capacity, albeit to a limited extent. Complete loss of p53 abrogates both mechanisms of MET downregulation, resulting in its highest expression. Despite this, MET becomes active only after phosphorylation, which is induced by its ligand HGF, derived from stromal cells residing in the ovarian tumor surroundings [157]. Collectively, the data indicates that *TP53* mutation is sufficient for preprogramming epithelial cells’ motility and invasion, but the stromal response seems to be essential for their manifestation through tumorigenesis [157].

Taken together, these results suggest that p53 plays an important role in the interplay between HGSC cells and fibroblasts in microenvironment on both sides - the mutated p53 in cancer cells and the WT p53 in CAFs, that is either down regulated or drives an altered transcriptional program. 

The immune system and inflammatory response are another important component of the tumor microenvironment interaction. Ovarian cancer is strongly associated with inflammation [160,161,162]. The p53 tumor suppressor signaling pathway is engaged in critical aspects of tumor immunology, immune responses, and inflammation [163,164,165,166], for instance, by directly activating expression of immunity-responsive genes, such as interferons and chemokines [167,168]. *TP53* mutations play a key role in switching inflammation effects to oncogenic outcomes so that cancer cells become more aggressive in response to inflammatory cytokines [169]. Chemokines and their receptors have been implicated in OC progression and metastasis [170,171]. It has been previously demonstrated that loss of p53 in EOC leads to a proinflammatory response via TNFα and NF-κB signaling, which in-turn promotes tumor progression [172,173]. The same research group has also identified the presence of a distinct chemokine signature in ovarian carcinomas bearing WT *TP53* and mutant *TP53*, and its correlation with better or worse overall survival. Mutant *TP53* serous EOC had higher expression levels of CCL8, CCL20, CXCL10, and CXCL11 compared to WT *TP53,* albeit this difference can also be attributed to WT *TP53* likely representing low grade serous ovarian cancer and mutant *TP53* representing HGSC [172].

Overexpression and nuclear accumulation of mutant p53 triggers an endogenous immune response that leads to generation of anti p53 auto-antibodies (AAbs) [174]. p53-AAbs may induce amplification of p53-specific T cell immunity, which has been detected in tumor-infiltrating lymphocytes in ovarian cancer [175]. About 60% of HGSC tumors carry missense *TP53* mutations, leading to expression of an aberrant protein, yet for an unknown reason only approximately 22–25% of HGSC cases were found to carry p53-AAbs [176]. Nevertheless, p53-AAbs levels are significantly higher in women with high-grade (type II) serous carcinomas as compared to women with low grade (type I) serous carcinomas or healthy individuals [177]. Additionally, HGSC patients carrying any *TP53* mutations had considerably higher p53-AAbs levels than those with WT *TP53*, regardless of the exon in which the mutation resides [177]. Contradictory findings have been reported in respect to the prognostic relevance of p53-AAbs in HGSC patients. While a number of studies showed no change in outcome [177,178,179,180], others indicated better [181,182] or worse [183,184] prognosis (Table 3).

The presence of p53-AAbs in ovarian cancer for a diagnostic purpose has been the focus of many studies. Yang et al. showed that in advanced EOC patients, p53-AAbs levels can be elevated 8 months prior to CA125 and 22 months prior to clinical diagnosis in patients who do not experience a rise in CA125, emphasizing the importance of p53 as a marker that could complement CA125 at the time of diagnosis [176]. Three other studies showed a potential for p53 AAbs for early EOC diagnosis [185,186,187]. However, data from a study involving 194 women with OC that aimed to estimate the diagnostic capacity of p53 AAbs for invasive EOC, indicated that the added value of p53-AAbs as a marker for early detection of OC is still limited [188].

## 8. p53 as a Target for EOC Therapy

The high frequency of p53 alterations in HGSC, coupled with the low frequency of mutations in normal organs, makes p53 an attractive target for HGSC specific therapy. The shared p53 mutation in all cancer cells, resulting from its early appearance during transformation, overcomes the common problem encountered in most targeted therapies - intra-tumoral heterogeneity. However, although the understanding that p53 is a plausible target for therapy has been entertained for many years [189], the first p53 targeting agents have reached clinical trials only in recent years [190] and no p53 targeting agents have been approved to date. The main caveat in targeting p53, similar to other transcription factors, is that p53 does not harbor a catalytic domain that would allow targeting the protein with small molecule compounds. Additionally, the nuclear localization of p53 hinders targeting p53 with monoclonal antibodies, therefore making p53 essentially “non-druggable” for many years. 

The most advanced efforts in targeting mutant p53 have been in restoring p53 native function. p53 structure mutants harbor a conformation that cannot perform the tumor suppressive activity and can have a dominant negative oncogenic role [191]. Therefore, a compound that would restore the native WT structure would not only inhibit the oncogenic role, but could potentially restore the original tumor suppressive role. Several compounds aimed to restore the original p53 conformation; the most advanced of this type was APR-246. APR-246 is metabolized to the active compound methylene quinuclidinone (MQ), which is a Michael acceptor which reacts with Cysteine residues in mutant p53 and converts it back to the WT structure [192]. APR-246 was tested in a phase 1 trial in patients with relapsed platinum sensitive HGSC in combination with carboplatin and PLD. This trial showed a 78% response rate and tolerable side effects profile, with dizziness being the most common adverse event (AE) attributed to APR-246 [193]. A phase 2 trial of APR-246 and PLD was conducted in platinum resistant HGSC, but the results have not yet been published [194].

Several hotspot p53 mutants have been shown to aggregate and induce aggregation also of wild type p53, leading to an oncogenic gain of function, and inhibition of the tumor suppressive activity of WT p53 [195]. Therefore, inhibition of mutant p53 aggregation can potentially restore wild type p53 activity. Several attempts have been made to inhibit mutant p53 aggregation in vitro. Specifically, small stress molecules such as polyarginine, polyornithine, and acetylcholine chloride inhibited growth of p53 mutant cancer cells and restored the transcription of the well-known WT p53 target gene CDKN1A (encoding for p21) [196,197]. Another effort to inhibit mutant p53 aggregation was performed by a designer peptide termed ReACp53 [198]. In HGSC cell lines, organoids and xenografts, treatment with ReACp53 inhibits mutant p53 aggregation, restores WT p53 activity, and inhibits cancer cell growth [198]. However, the ReACp53 also has disadvantages; mainly that it acts only on cells with mutations in two specific p53 residues, R175 and R248. 

Another method for WT p53 reactivation that was widely explored, including in clinical trials, is inhibition of p53 degraders such as MDM2, MDM4 and MDMX [199,200]. However, this method of p53 activity restoration is relevant in p53 WT tumors, and therefore not applicable in HGSC. 

An entirely different approach towards mutant p53 targeting is using it to activate an anti-cancer immune response. The earlier attempts that have already reached clinical trials include the use of viral vectors carrying the wild-type *TP53* gene. Ad-p53 (Gendicine) is an adenovirus based viral vector coding for WT p53, used for intra-tumoral injections. This vector was approved by the China Food and Drug Administration (CFDA) in 2003 as gene therapy for Head and Neck cancer [201]. It has since been tested in clinical trials for ovarian cancer in intraperitoneal injections in combination with chemotherapy, but failed to show an advantage over chemotherapy alone [202]. Another viral vector carrying WT *TP53* that has reached clinical trials is the modified vaccinia virus Ankara vaccine expressing p53 (p53MVA). P53MVA was tested in a phase 1 trial in combination with Gemcitabine and showed a lower response rate than what is expected for Gemcitabine alone [203]. However, it is currently being tested in combination with the PD-1 inhibitor pembrolizumab in a phase 2 clinical trial for treatment of ovarian cancer [204]. 

A different approach to trigger an immune response towards p53 has recently been successful in pre-clinical results. This approach stems from the finding that mutant p53 undergoes proteolytic cleavage at the proteasome, and fragments are presented on major histocompatibility complex molecules [205]. Since p53 mutations are often missense mutations, the proteolysis leads to generation of abnormal peptides that illicit an immune response when presented on HLA molecules [205,206]. Presentation of cancer neoantigens on HLA complexes are extensively studied as a target for immune activation in different cancer types [207,208]. Lo et al. isolated T-lymphocytes from a colon cancer patient carrying a T-cell receptor recognizing mutant p53 R175H and presenting on HLA-A*0201 molecules. These T-lymphocytes reacted in vitro against commercial cell lines, including ovarian cancer cell lines, carrying the R175H mutant in the context of HLA-A*0201. However, generating T-lymphocytes is a cumbersome and very specific method, tailored individually to each patient with their own T-lymphocytes. An easier way to target cancer neoantigens is using bi-specific antibodies that target T-lymphocytes (e.g., CD3) and the neoantigen – bringing them both in close proximity and thus facilitating an interaction between the lymphocyte and the neoantigen. Recently Hsiue et al. [209] generated bi-specific antibodies that targeted mutant p53 R175H and CD3, which have been shown effective in targeting cancer cells, including the ovarian cancer cell line TYK-nu in vitro and in vivo in mice. These mutant p53 targeting techniques have not been tested in patients to the best of our knowledge, but we expect these experiments to follow the in vitro and mouse experiments.

However, the caveats of using p53 as a neoantigen presented on HLA molecules is the specificity of this therapy to so far only R175H mutants, which according to the TCGA are only about 3.4% of HGSC. Furthermore, this therapy is currently restricted to HLA-A*0201, which is shared by only 40% of the Caucasian population of the USA, further restricting the usability of this method. Another caveat is that T-cell therapy or bispecific T-cell engaging antibodies have not yet proven effective in solid tumors. Nevertheless, this new method is a promising approach that has the potential to be a highly specific and personalized therapy for HGSC patients. 

## 9. Conclusions

p53 mutations are the diagnostic hallmark of HGSC, and as detailed above, mutant p53 governs many aspects of cancer initiation and progression. Despite many studies on the role of mutant p53 in HGSC, there is still a discrepancy between the key role of p53 in this disease and the very few recognized mutant p53 target genes. This suggests there are likely additional roles of p53 that remain unknown. Since research into targeting of p53 is making progress, and might become a reality in the near future, likely resistance to p53 targeted therapy will emerge. Better understanding of mutant p53 mechanism of action would enable overcoming resistance to p53 targeted therapeutics. 

## Figures and Tables

**Table 1:** Regulators of mutant p53 function.

Gene Symbol	Function	Effect on p53 in HGSC	Reference
PAX8	Transcription factor	Transcriptionally upregulates GOF p53	[92]
TNFRSF12A	Member of the tumor necrosis factor receptor super-family	Enhances MDM2 mediated ubiquitination and degradation of R248Q mutant p53	[93]
TRIM-71	E3 ubiquitin ligase	Induces degradation of p53 mutants	[94]
USP15	Deubiquitinase	Elevates levels of the aggregated GOF p53 R175H	[95]
MEG3	lncRNA	Upregulates mutant p53 RNA and protein expression levels	[96]
miR let-7d-5p	microRNA	Affects mutant p53 expression in the mRNA and protein level through targeting HMGA	[97]

**Table 2:** Downstream targets of p53 in HGSC.

p53 Downstream Effectors	Regulation by p53	Biological Effect	Reference
CDKN1A (p21)	Direct transcriptional regulation by mutant p53	Oncogenic (resulting from an oncogenic role of p21)	[92]
PLAC1	Direct transcriptional de-repression by mutant or null p53 of the inhibitory effect of WT p53	Oncogenic function	[107]
Notch3	Transcriptional upregulation by mutp53	Activates CCNG1 and leads to promoted HGSC metastasis and cisplatin resistance	[108]
Target genes of β-arrestin1/YAP/mutp53 complex	Gene-specific transcriptional regulation	Enhanced proliferation, survival, and invasion of HGSC in vitro and metastasis in vivo	[109]
ACP6	Downregulation by p53 mutants R175H, R249S and R273H	Increased LPA levels and increased migration and proliferation	[110]

**Table 3:** The role of p53 in the microenvironment.

Pathway	Cell Expressing p53	Biological Effect	Reference
p53	Cancer associated fibroblasts	Changes in the transcriptional signature from a tumor suppressive to a tumor supportive signature	[155]
IL-1β	Cancer associated fibroblasts	Secretion from epithelial cancer cells leads to down regulation of WT p53 in CAFs	[155]
ICAM1	Cancer associated fibroblasts	Knockdown of WT p53 from CAF induces an ICAM1 mediated invasiveness of immortalized epithelial ovarian cells	[156]
MET	Tumor cells	Mutant p53 upregulates Met, that is a receptor to stromal cell derived HGF	[157]
NFκB, TNFα	Tumor cells	loss of p53 in EOC leads to a pro-inflammatory response	[172,173]
Immune activation	Tumor cells	Mutant p53 in HGSC leads to generation of anti p53 auto-antibodies and p53 specific T-cell immunity	[174,175,176,177,178,179,180,181,182,183,184]

## Data Availability

Not applicable.
